# Molecular Competition: Flame Retardants Interact with Key Metabolism Enzyme

**DOI:** 10.1289/ehp.121-A313

**Published:** 2013-10-01

**Authors:** Kellyn S. Betts

**Affiliations:** Kellyn S. Betts writes about environmental contaminants, hazards, and technology for solving environmental problems for publications including *EHP* and *Environmental Science & Technology*.

Using X-ray crystallography to visualize the three-dimensional structure of an enzyme associated with regulating levels of estrogen, a team of National Institutes of Health scientists have discovered new information on how flame retardants may alter estrogen metabolism.^1^ Their results suggest one way in which these chemicals may disrupt the body’s endocrine system.

Flame retardants are added to foam, textiles, electronics, building materials, and other items to reduce flammability.^2^ The new research focuses on tetrabromobisphenol A (TBBPA), currently the most heavily produced flame retardant in the world, and 3-OH-BDE-47, a metabolite of the Penta bromodiphenyl ethers (PentaBDEs), which were widely used prior to 2004 and remain in many long-lived consumer goods.

Flame retardants come in two types: reactive agents, which are chemically bound to base materials during manufacturing, and additive agents, which are incorporated into products but not chemically bound, allowing them to escape into the surrounding environment.^3^ People are exposed mainly to additive flame retardants that have migrated into house dust,^4^ with children potentially more exposed than adults because of their more frequent hand-to-mouth activity and contact with floors.^5^^,^^6^ PentaBDEs were used as additive flame retardants, and although TBBPA is mainly used as a reactive agent, it can also be used as an additive retardant.

The new research shows how both TBBPA and 3-OH-BDE-47 can bind to an enzyme known as estrogen sulfotransferase (SULT1E1). This enzyme’s job is to bind the major endogenous estrogen 17β-estradiol and add a sulfate molecule to it; the sulfated estradiol is more readily eliminated from the body. “Basically, the flame retardants are going to compete with estradiol for binding to the sulfotransferase,” says coauthor Linda Birnbaum, director of the National Institute of Environmental Health Sciences (NIEHS). The result may be to raise levels of estradiol in the body, explains corresponding author, Lars Pedersen of the NIEHS Laboratory of Structural Biology.

**Figure 1:**
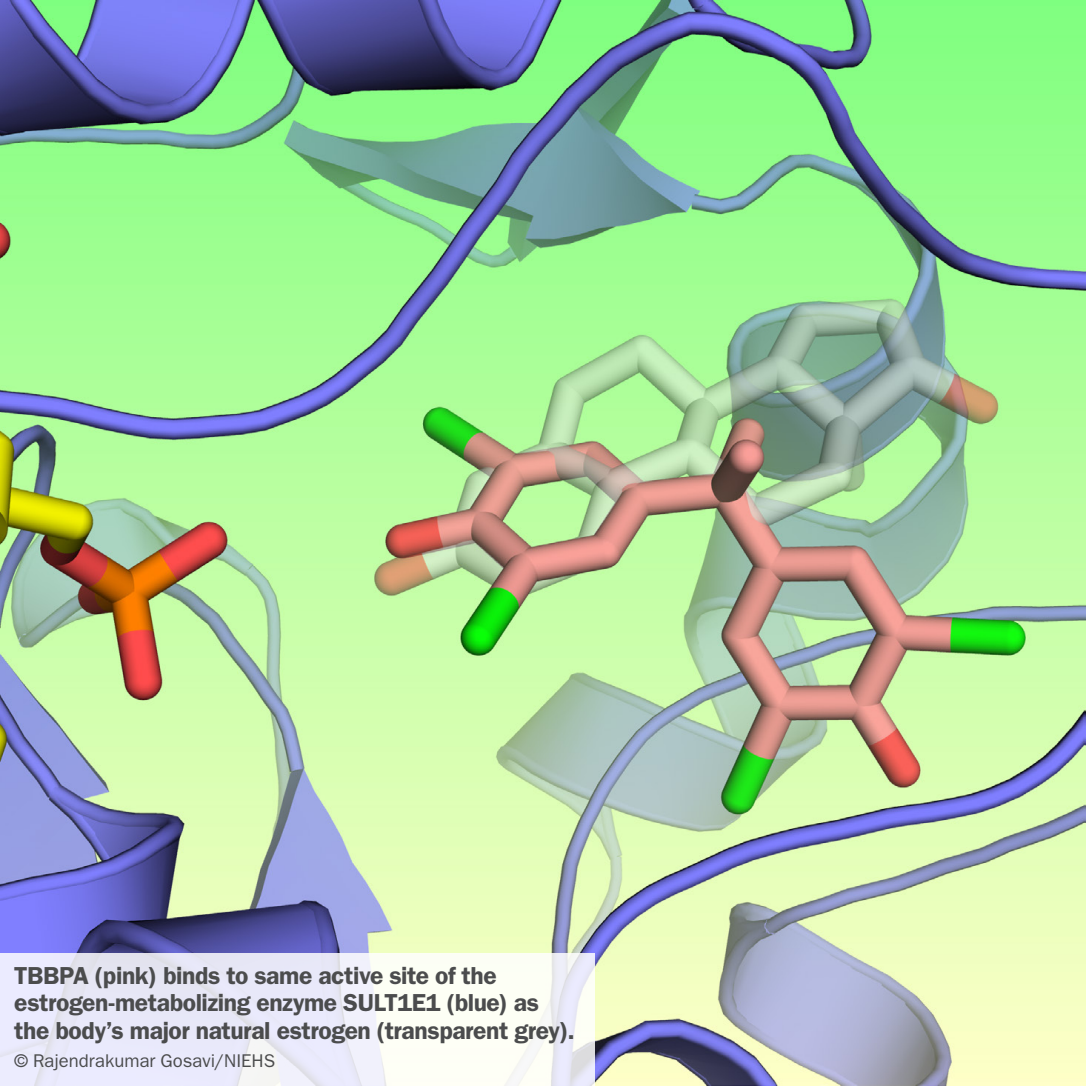
TBBPA (pink) binds to same active site of the estrogen-metabolizing enzyme SULT1E1 (blue) as the body’s major natural estrogen (transparent grey). © Rajendrakumar Gosavi/NIEHS

Endocrine disruption can take several forms—transport disruption, receptor binding, and metabolism are all processes that will affect the endocrine system, Pedersen says. This research demonstrates a potential metabolic impact on the endocrine system and shows that xenobiotic compounds don’t have to bind to the hormone receptor in order to perturb the system, Birnbaum points out.

Pedersen adds that these structures only show how these chemicals inhibit the enzyme in the lab. “We need to figure out if this is what is going on in the body,” he says.

Given the structural differences between 17β-estradiol and the flame retardants, Pedersen says he was surprised the exogenous molecules were able to bind SULT1E1. Craig Butt, a postdoctoral environmental chemist at Duke University’s Nicholas School of the Environment, observes, “It shows that there is a little bit of flexibility in terms of what molecules can fit into the enzyme, which explains why these foreign chemicals can inhibit the action of the enzyme.”

The new research raises concerns because hormones are designed to regulate key mechanisms in our bodies, and they do this at very low levels in our bloodstream and tissues, says Heather Stapleton, an associate professor of environmental chemistry at Duke and Butt’s advisor. “Having continuous exposure to man-made chemicals that can bind with the same affinity as endogenous hormones may have very significant consequences on development and overall health,” Stapleton says.

Birnbaum agrees: “We know that hormones are important in many, many aspects of our lives. … One of the important things is, it’s not a single hormone that may be critical. Often it’s the balance of multiple hormones. We all have our own balance. It’s possible you can throw one person out of balance by perturbing one system while in another person you would not.”

## References

[1] Gosavi RK Mimicking of estradiol binding by flame retardants and their metabolites: a crystallographic analysis. Environ Health Perspect 121 10 1194 1199 2013; http//dx..org/10.1289/ehp.1306902. 23959441 PMC3801471

[2] Shaw SD Halogenated flame retardants: do the fire safety benefits justify the risks? Rev Environ Health 25 4 261 305 2010; http//dx..org/10.1515/REVEH.2010.25.4.261. 21268442

[3] Alaee MA An overview of commercially used brominated flame retardants, their applications, their use patterns in different countries/regions and possible modes of release. Environ Int 29 6 683 689 2003; http//dx..org/10.1016/S0160-4120(03)00121-1. 12850087

[4] Stapleton HM Polybrominated diphenyl ethers in house dust and clothes dryer lint. Environ Sci Technol 39 4 925 931 2005; http//dx..org/10.1021/es0486824. 15773463

[5] Stapleton HM Serum PBDEs in a North Carolina toddler cohort: associations with handwipes, house dust, and socioeconomic variables. Environ Health Perspect 120 7 1049 1054 2012; http//dx..org/10.1289/ehp.1104802. 22763040 PMC3404669

[6] Rose M, Bennett DH, Bergman A, Fangstrom B, Pessah IN, Hertz-Picciotto I (2010). PBDEs in 2–5 year-old children from California and associations with diet and indoor environment.. Environ Sci Technol.

